# First Identification and Genetic Characterization of a Novel Duck Astrovirus in Ducklings in China

**DOI:** 10.3389/fvets.2022.873062

**Published:** 2022-04-08

**Authors:** Junqin Zhang, Yunzhen Huang, Linlin Li, Jiawen Dong, Ruihuan Kuang, Ming Liao, Minhua Sun

**Affiliations:** ^1^Institute of Animal Health, Guangdong Academy of Agricultural Sciences, Guangzhou, China; ^2^Key Laboratory of Avian Influenza and Other Major Poultry Diseases Prevention and Control, Ministry of Agriculture and Rural Affairs, Guangzhou, China; ^3^Key Laboratory of Livestock Disease Prevention and Treatment of Guangdong Province, Guangzhou, China; ^4^Scientific Observation and Experiment Station of Veterinary Drugs and Diagnostic Techniques of Guangdong Province, Ministry of Agriculture, Guangzhou, China; ^5^Guangdong Academy of Agricultural Sciences, Guangzhou, China

**Keywords:** duck astrovirus, gout, genome sequence, phylogenetic analysis, genetic distance

## Abstract

Four divergent groups of duck astroviruses (DAstVs) have been identified that infect domestic ducks. In March 2021, a fatal disease characterized by visceral urate deposition broke out in 5-day-old Beijing ducks on a commercial farm in Guangdong province, China. We identified a novel duck astrovirus from the ducklings suffering from gout disease. The complete genome sequence of this DAstV was obtained by virome sequencing and amplification. Phylogenetic analyses and pairwise comparisons demonstrated that this DAstV represented a novel group of avastrovirus. Thus, we designated this duck astrovirus as DAstV-5 JM strain. DAstV-5 JM shared genome sequence identities of 15–45% with other avastroviruses. Amino acid identities with proteins from other avastroviruses did not exceed 59% for ORF1a, 79% for ORF1b, and 60% for ORF2. The capsid region of JM shared genetic distances of 0.596 to 0.695 with the three official avastrovirus species. suggesting that JM could be classified as a novel genotype species in the Avastrovirus genus. Meanwhile, JM shares genetic distances of 0.402–0.662 with all the other known unassigned avastroviruses, revealing that it represents an additional unassigned avastrovirus. In summary, we determined that the DAstV-5 JM strain is a novel genotype species of avastrovirus.

## Introduction

Astroviruses (AstVs) belong to the *Astroviridae* family and are infectious, non-enveloped, positive sense, single-stranded RNA viruses. Astroviruses infect a wide variety of hosts. According to their host range, the *Astroviridae* family is divided into two genera: *Mamastrovirus* and *Avastrovirus*. The two genera comprises 22 species according to the International Committee on Taxonomy of Viruses (ICTV), namely *Mamastroviruses* 1–19 and *Avastroviruses* 1–3 (1). The *Avastroviruses* genus include *Avastrovirus 1* (turkey astrovirus 1), *Avastrovirus 2* (avian nephritis virus 1 and avian nephritis virus 2) and *Avastrovirus 3* (turkey astrovirus 2 and duck astrovirus 1). These *Avastroviruses* are tightly linked to avian diseases, such as hepatitis (2), enteritis (3), runting-stunting syndrome (4), white chicken syndrome (5), and gout (6). Besides, there are many other newly discovered genotypes of astroviruses that have not been classified into any *Avastrovirus* species, but they have been proposed to the ICTV, referring as unassigned species.

To date, chicken astrovirus and goose astrovirus have been found to cause avian gout diseases. Chicken astrovirus was reported as the causative agent of chicken gout in India and Malaysia (7–9). Goose astrovirus was observed to cause fatal visceral gout in domestic goslings in China since 2017 (10). Goose astroviruses isolated from ducks could also cause gout in ducklings (11–13). In addition, goose astrovirus has been validated to cause gout in experimentally infected chickens (14). However, no case of duck astrovirus-induced gout in ducks has been reported. Up till now, four genotypes of duck astroviruses have been identified: duck astrovirus 1 (DAstV-1), duck astrovirus 2 (DAstV-2), duck astrovirus 3 (DAstV-3), and duck astrovirus 4 (DAstV-4). These four duck astroviruses are genetically different from each other. DAstV-1 and DAstV-2, also known as duck hepatitis 2 (DHV-2) and duck hepatitis 3 (DHV-3), cause hepatitis in ducklings (2). DAstV-3 was first found in newly hatched, healthy Pekin ducklings in 2013 (15). DAstV-4 was isolated from Pekin ducks in live-bird markets in Guangdong province (16). However, no disease has been reported to be caused by DAstV-3 and DAstV-4.

In the present study, we discovered a new duck astrovirus DAstV-5 in ducklings suffering from gout disease. We conducted virome sequencing and obtained the complete genome sequence of the virus. Phylogenetic analyses and pairwise comparisons demonstrated that this virus was different from all the other previously known astroviruses, and thus we proposed it as a novel genotype species of duck astrovirus.

## Materials and Methods

### Bacteriological and Virological Examinations

For bacteriological diagnosis, kidney and liver tissues were collected from dead ducklings and inoculated onto tryptic soy agar plates containing 2% fetal bovine serum (Hyclone, Shanghai, China) immediately. The plates were incubated at 37°C with 5% CO_2_ for 48 h. For virological detection, kidney and liver tissue samples were homogenized in phosphate-buffered saline (PBS) and pooled together for three cycles of freezing and thawing. The suspensions were centrifuged at 12,000 × *g* for 10 min and then the supernatant was collected for virus RNA/DNA extraction using a Tianlong Nucleic Acid Extraction & Purification Kit T180H (Tianlong Technology Co., Ltd., Shanxi China). The extracted RNA/DNA was subjected to polymerase chain reaction (PCR) amplification to detect goose astrovirus (GAstV), avian orthoreovirus virus (ARV), duck Tembusu virus (DTMUV), novel duck parvovirus (NDPV), duck circovirus (DCV), goose parvovirus (GPV), duck enteritis virus (DEV), duck adenovirus (DAdV), avian influenza virus (AIV), and Newcastle disease virus (NDV). The primers used to amplify the genome sequences were detailed to our previous research (17, 18) and are shown in Supplementary Table 2.

### Virome Sequencing and Analysis

Five grams liver tissue was ground. After three rounds of freeze-thawing, the tissue was centrifuged at 12,000 g for 5 min to remove the precipitate. The supernatant was centrifuged at 160,000 g for 2 h at 4°C with HIMAC CP 100wx ultracentrifuge (Hitachi, Tokyo, Japan). The supernatant was used to extract DNA/RNA with an R6662-02 MagPure Viral DNA/RNA Mini LQ Kit (R6662-02, Angen Biotech, Guangzhou, China). The whole genome was amplified using REPLI-g Cell WGA & WTA Kit (150054, Qiagen, Germantown, MD, USA). Sequencing libraries were generated using an NEB Next® Ultra™ DNA Library Prep Kit for Illumina® (New England Biolabs, Ipswich, MA, USA) following the manufacturer's recommendations. The library was sequenced on an Illumina Novaseq 6,000 and 150 bp paired-end reads were generated. The raw data were processed using SOAPnuke (v1.5.6) to acquire the clean data for subsequent analysis (19).

Firstly, we used BWA software (v0.7.17) to compare the clean reads with ribosome database (Silva.132) and host database respectively. If the comparison length was <80% of the total length of reads, the reads were filtered. Then the host sequences were removed. The BWA software (v0.7.17) was used to compare the clean reads to virus reference database (Virus-NT), And we filtered the comparison results whose comparison length was <80% of the total length of reads for preliminary virus classification. Secondly, we used Megahit software (v1.1.2) to assemble the clean data into contigs. The contigs that compared to the host sequence database by BLAST software (v2.9.0+). And the host sequences were removed. Finally, we used BLAST software (v2.9.0+; NIH, Bethesda, MD, USA) to compare the unique contigs with virus database (separated from the NT database). If the alignment similarity was ≥80%, the alignment length was ≥500 bp, and e ≤1e-5, the contig was defined as virus sequence. If the alignment length was ≥100 bp, and e ≤1e-5, the contig was defined as suspected virus sequence. After comparing contigs with the virus database obtained from the NT database and the virus database obtained from the NR and HMM (vpfs and vfam) databases, we obtained the consensus sequences as the candidate virus sequence. The virome sequencing and data analysis were performed by Magigene Biotechnology Corporation (Guangzhou, China). The candidate virus sequence alignments were performed in NCBI Nucleotide BLAST site (https://blast.ncbi.nlm.nih.gov/Blast.cgi). The authenticity and similarity of the sequences were validated. The most homologous virus strain was used as a reference strain. The sequence was assembled by using reference mapping.

### Obtaining Complete Genome Sequence of the Virus

Four pairs of PCR primers were designed to amplify the gaps (Supplementary Table 3) using 2 × Taq Plus Master Mix (Vazyme, Jiangsu, China). The four amplified segments were ligated to the pGEM-T vector and sequenced. The 5' and 3' untranslated regions (UTRs) were obtained using 5' and 3' rapid amplification of cDNA ends (RACE) as described previously (20). To verify the complete genome sequence, we designed eight pairs of primers. The primers used to amplify the genome sequences are shown in Supplementary Table 3. The complete genome sequence was assembled by DNAMAN 6.0.3 (Lynnon Biosoft, San Ramon, CA, USA). And DAstV CPH strain was used as a reference strain to assemble the complete genome sequence.

### Sequence Analysis

Transmembrane (TM) domains and functional proteins were predicted using the Sample Modular Architecture Research Tool (SMART) analysis tool https://smart.embl-heidelberg.de/. The stem-loop II-like motif (s2m) was evaluated using the online Rfam analysis tool (http:/rfam.xfam.org). The open reading frames (ORFs) were analyzed by DNAMAN version 8.0 (Lynnon Biosoft). Phylogenetic trees for the complete genome and the deduced amino acid sequences of the three open reading frames (ORFs) were constructed in the MEGA 7.0 software using the neighbor-joining method, with 1,000 bootstrap replications (21). Amino acid genetic distances were analyzed using the *p*-dist method of MEGA 7.0 software, using pairwise deletion treatment. Bootstrap analysis was performed with 100 replications. Pairwise comparisons of DAstV-5 JM with other avastroviruses were conducted using the MegAlign 7.1.0 software (DNASTAR Inc., Madison WI, USA) employing the Clustal W method.

### Genetic Recombination Analysis

Recombination event analysis of DAstV-5 JM with other astroviruses (including *Mamastroviruses* and *Avastrovirus*) was evaluated using Recombination Detection Program (RDP) software Version 4.101 with the RDP, GENECONV, Bootscan, MaxChi, Chimaera, SiScan, and 3Seq methods (22).

## Results

### Case History and Microbiological Examination

**Figure 1:**
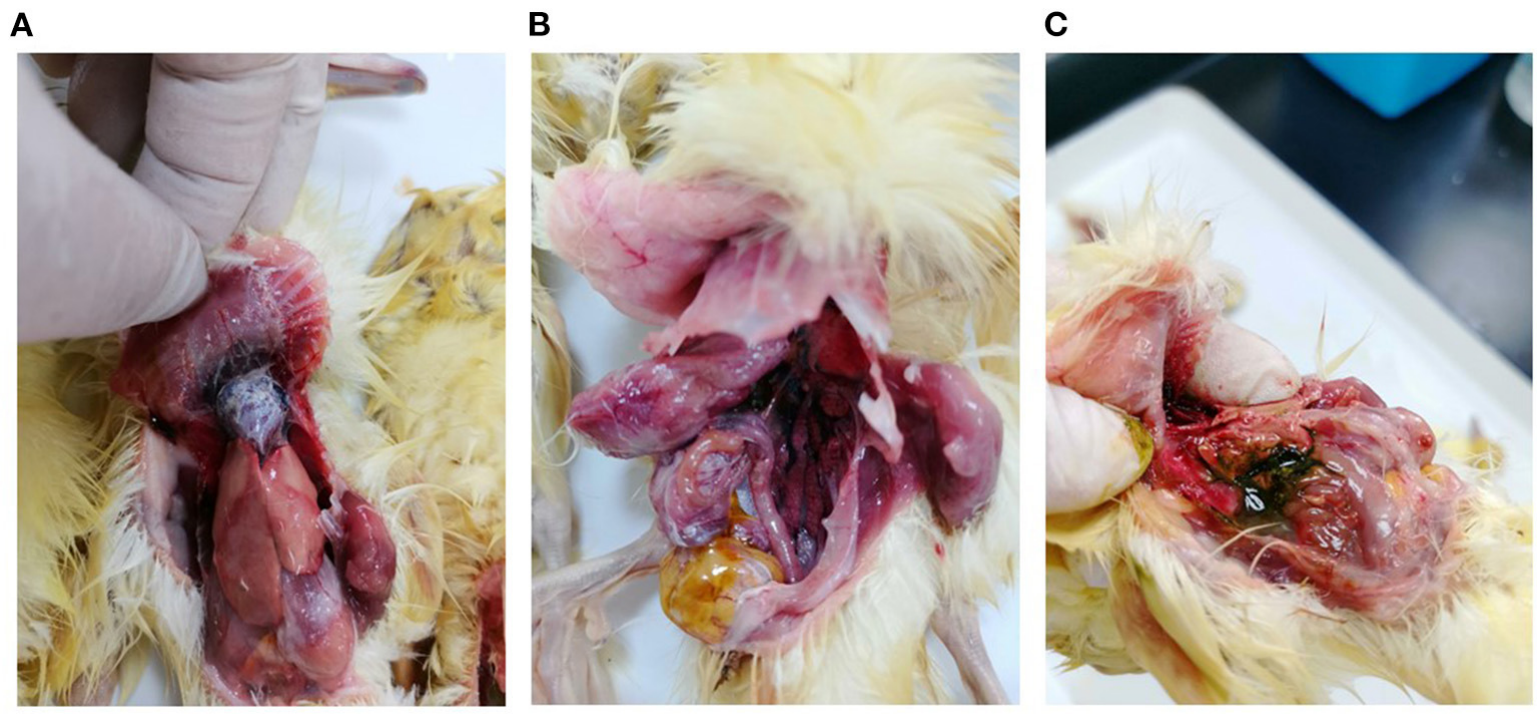
Pathological lesions of clinical samples. **(A)** Urate deposition in the heart; **(B)** Urate deposition in the mesenterium; **(C)** Urate deposition in the gallbladder.

In March 2021, a gout disease broke out in 5-day-old Beijing ducks on a commercial farm in Guangdong province, China. The mortality rate was 20% on this farm. At necropsy, urate deposits were observed mainly in the heart, mesenterium, and gallbladder of the dead ducklings (Figure 1). Bacteriological examination was conducted as described previously (6), and no pathogenic bacteria could be isolated from the diseased ducklings. The DNA/RNA were extracted from the liver and heart tissues and the isolated nucleic acids tested negative for GAstV, ARV, DTMUV, NDPV, DCV, GPV, DEV, DAdV, AIV, and NDV by PCR assays.

### Complete Genome Sequence of DAstV-5 JM Strain

To identify the pathogen, we carried out virome sequencing analysis. Through high-throughput sequencing, 20,627,440 clean reads were identified. After removing ribosomal sequences and host sequences, 1,398 reads were virus sequences among these clean reads. Then the 1,398 reads were assembled into 307 contigs using Megahit (version 1.1.2). Among these 307 contigs, 153 contigs were phage sequences and the rest 154 contigs were other virus sequences. Then we put these 154 contigs separately into the online NCBI Nucleotide BLAST site (https://blast.ncbi.nlm.nih.gov/Blast.cgi). Finally, we identified that 6 contigs are virus sequences. The other 148 contigs are identified as bacterial sequences and host sequences. The six virus sequences include five duck astrovirus sequences and one chicken stool-associated gemycircularvirus sequence. The chicken stool-associated gemycircularvirus sequence was 741 bp in length. And the astrovirus sequences were 253 bp to 1,006 bp in length. The astrovirus segments were homologous to previously published nucleotide sequences. And the sequence similarities were 52–77% (Supplementary Table 1). We chose the most homologous strain, DAstV CPH strain, as a reference strain for mapping. Specifically, we compared the identified five astrovirus sequences with DAstV CPH strain's whole genome sequence. Then the positions of these sequences in the viral genome are basically determined. And the primers for amplifying the gaps were designed between the two adjacent sequences. Then the four gaps between these five segments were amplified. The presence of astrovirus (designated JM) was confirmed by PCR amplification.

To obtain the whole genome sequence of this duck astrovirus JM strain, the sequences of five segments, four gaps, and two UTRs were assembled together. Finally, we obtained a 7,517 bp viral genome sequence of JM strain. The assembled whole genome sequence was verified by PCR amplification and sequencing. Eight pairs of primers used here were showed in the Supplementary Table 3. Alignment results showed that the amplified sequences were in accordance with the assembled whole genome sequence. The complete genomic sequence of the virus, named as DAstV-5 JM strain, has been submitted to the GenBank database under the accession number OM095382.

### Genetic Characteristics of the DAstV-5 JM Strain

**Figure 2:**
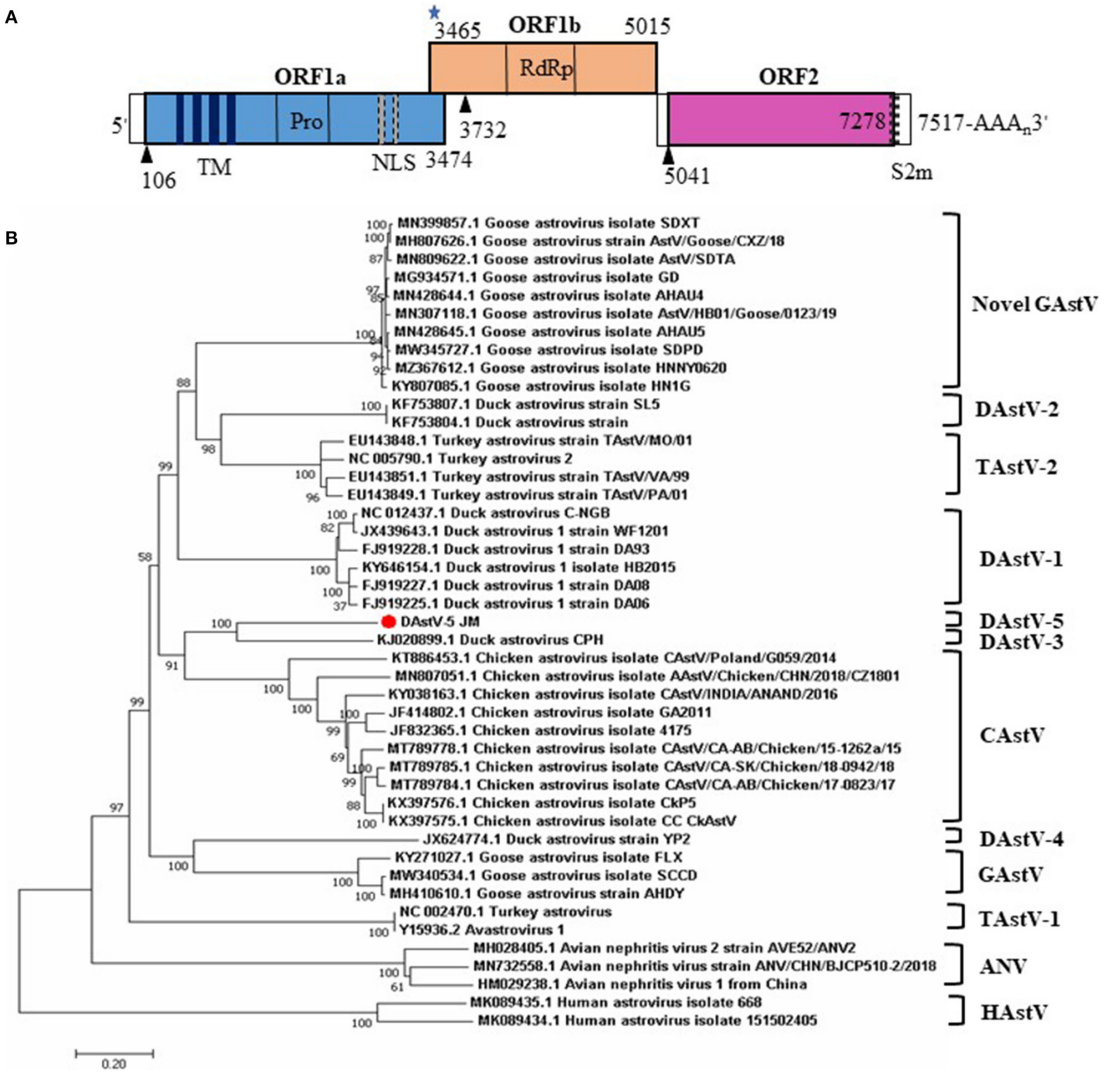
Genome organization and phylogenetic analysis of the complete genome sequences of DAstV-5 JM strain. **(A)** The three predicted ORFs and the typical genomic characteristics are shown. The black triangles represent the translation start site of the three ORFs. The blue star represents the heptameric AAAAAAC sequence. **(B)** The phylogenetic tree was constructed using MEGA 7.0 software using the neighbor-joining method, with 1,000 bootstrap replications. ANV, avian nephritis virus; CAstV, chicken astrovirus; DAstV, duck astrovirus; GAstV, goose astrovirus; HAstV, human astrovirus; TAstV, turkey astrovirus.

The predicted genomic organization of DAstV-5 JM is shown in Figure 2A. The genomic length of DAstV-5 JM is 7517 nt, including a 5' UTR, ORF1a, ORF1b, ORF2, 3' UTR, and poly (A) tail. ORF1a and the ORF1b encode the non-structural proteins, with a calculated molecular weight of 128.62 kDa. ORF1a is not in the same reading frame with ORF1b. As predicted by the online SMART analysis tool, the ORF1a protein contains four transmembrane (TM) domains, a trypsin-like serine protease, and two nuclear localization signal (NLS) motifs. ORF1b was predicted to encode an RNA-dependent RNA polymerase (RdRp), with a calculated molecular weight of 50.27 kDa. It overlaps by 10 nt with ORF1a. It starts with a ribosomal frameshift signal containing the heptameric AAAAAAC sequence (3,465–3,471) and contains a stem loop structure sequence (3,478–3,501). ORF2 encodes the capsid protein, with a calculated molecular weight of 82.42 kDa. The start codon of ORF2 is 25 nt downstream of the stop codon of ORF1b; therefore, ORF2 of DAstV-5 JM is not in the same reading frame as ORF1b. A CCGAA motif (5027-5031) was identified within the 25 nt sequence. Like other astroviruses, a stem-loop, s2m, is also present at the end of ORF2 and the adjacent 3' UTR (7,255–7,297). Taken together, these results indicated that DAstV-5 JM possesses typical astrovirus features.

### Phylogenetic Analyses and Sequence Comparisons of the JM Strain

**Figure 3:**
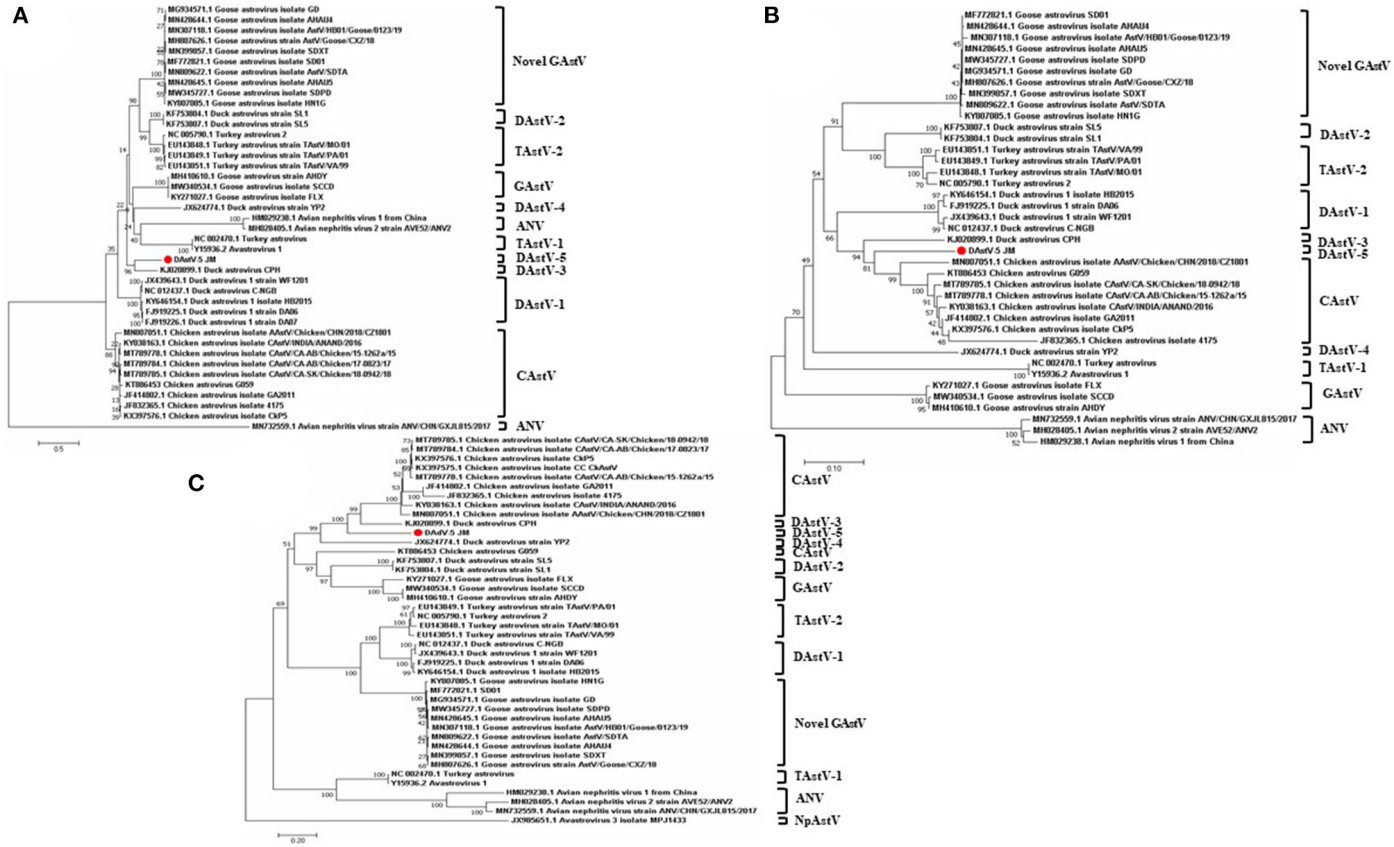
Phylogenetic analysis of the amino acid sequences of ORF1a **(A)**, ORF1b **(B)**, and ORF2 **(C)** of the DAstV-5 JM strain and other astroviruses. The phylogenetic trees were constructed using MEGA 7.0 software using the neighbor-joining method, with 1,000 bootstrap replications. DAstV-5 JM strain, Duck Astrovirus Virus-5 JM strain; ORF, open reading frame. ANV, avian nephritis virus; CAstV, chicken astrovirus; DAstV, duck astrovirus; GAstV, goose astrovirus; TAstV, turkey astrovirus.

To determine the phylogenetic relationships of duck astrovirus JM strain with other *Avastroviruses*, we conducted phylogenetic analysis of the complete genome of JM and the amino acid sequences of ORF1a, ORF1b, and ORF2. In the complete genome and the ORF1a amino acid phylogenetic trees, DAstV-5 JM strain was most closely related to DAstV CPH strain (Figures 2B, 3A). In the ORF1b and ORF2 amino acid phylogenetic trees, the DAstV-5 JM strain was closely related to both DAstV CPH strain and the chicken astrovirus clades (Figures 3B,C). Meanwhile, the DAstV-5 JM strain had an outgroup relationship with the DAstV CPH strain and the chicken astrovirus clades in the ORF1b and ORF2 amino acid phylogenetic trees.

Pairwise comparisons were performed to determine the sequence identities. The results showed that DAstV-5 JM shared identities of 15–45% in the complete genome sequences with the representative members of recognized and unassigned species in the genus *Avastrovirus* (Table 1). The sequence identities at the amino acid level were 26–59% (ORF1a), 55–79% (ORF1b), and 24–60% (ORF2). Although DAstV-5 JM is most closely related to DAstV CPH strain, they shared relatively low amino acid identities of 59% (ORF1a), 77% (ORF1b), and 60% (ORF2). According to the species demarcation criteria of the *Avastrovirus* genus from the ICTV, the mean amino acid genetic distances (*p*-dist) should range between 0.576 to 0.742, and 0.204 to 0.284 between and within groups, respectively (23). The results showed that the capsid region of JM shared a genetic distance of 0.596 to 0.695 with the representative strains of *Avastrovirus* 1–3. DAstV-5 JM shared genetic distances of 0.402, 0.440, 0.524, and 0.605 with DAstV CPH strain, chicken astrovirus GA2011, duck astrovirus YP2, and Duck astrovirus SL1 respectively. Therefore, DAstV-5 JM could be identified as a novel Avastrovirus.

**Table 1:** Comparison of complete genome sequences and amino acid sequences of the three ORFs of DAstV-5 JM strain with other representative strains of avastroviruses.

**Species**	**Virus**	**GenBank accession no**.	**Sequence identity (%)**				**Amino acid genetic distance (ORF2)**
			**Genome (nt)**	**ORF1a (aa)**	**ORF1b (aa)**	**ORF2 (aa)**	
*Avastrovirus 1*	Turkey astrovirus 1	Y15936	15	39	58	38	0.642
*Avastrovirus 2*	Avian nephritis virus 1	MN732559	20	26	55	29	0.695
*Avastrovirus 3*	Turkey astrovirus 2	NC_005790	31	47	68	42	0.596
	Duck astrovirus C-NGB strain	NC_012437	45	47	69	43	0.605
Unassigned	Duck astrovirus SL1 strain	KF753804	41	49	69	41	0.605
	Duck astrovirus CPH strain	KJ020899	43	59	77	60	0.402
	Duck astrovirus YP2 strain	JX624774	41	41	64	48	0.524
	Goose astrovirus SDXT strain	MN399857	29	48	68	36	0.662
	Goose astrovirus FLX strain	KY271027	21	48	65	37	0.641
	Chicken astrovirus Poland/G059/2014 strain	KT886453	26	54	79	43	0.586
	Chicken astrovirus GA2011 strain	JF414802	34	53	79	55	0.440
	MPJ1433/Northern pintail/091230 strain	JX985651	-	-	-	24	0.777

*DAstV-5, duck astrovirus 5*.

### Genetic Recombination Analysis

To detect the possibility of recombination events in DAstV-5 JM strain, we conducted genomic recombinant detection using RDP version 4.101 software. The analysis identified no recombination events between the JM strain and the other avian and mammalian astroviruses using the RDP, GENECONV, Bootscan, MaxChi, Chimaera, SiScan, and 3Seq methods.

## Discussion

Astroviruses are pathogenic to both humans and animals and have been detected from over 80 avian and mammalian host species (24). Astrovirus-induced gout disease has been prevalent in geese, chickens, and ducks in recent years, resulting in serious economic loses to the poultry industry. In this research, we identified a novel duck astrovirus, namely DAstV-5 JM, in the first recorded outbreak of deadly gout in ducklings.

According to the genetic organization analysis, the DAstV-5 JM strain possesses typical genomic characteristics: threes ORFs, TM domains, serine protease, NLS, ribosomal frameshift signal, and RdRp. Meanwhile, DAstV-5 JM strain has its own features. Avastroviruses possess four to six TM domains. DAstV-5 possesses four TM domains in ORF1a, as does DAstV-2, DAstV-3, and DAstV-4. However, DAstV-1 C-NGB strain, GAstV FLX strain, and GAstV HNNY0620 strain possess five TM domains (13, 25, 26). ANV-1 (ANV/CHN/BJCP510-2/2018) and CAstV-A (Poland/G059/2014) have six TM domains (27, 28). It appears that the number of TM domain is not related to the species of astrovirus. However, the function of the TM domains in astroviruses requires further exploration.

The DAstV-5 JM strain contains one s2m element at the end of ORF2 and the adjacent 3' UTR. Most astroviruses contains only one s2m element; however, several astrovirus, including CAstV GA2011, GAstV HNNY0620, and GAstV TZ03, contain two s2m elements, based on Rfam analysis (29). Notably, three Malaysian chicken astrovirus isolates contain three s2m elements (9). The s2m element is highly conserved, and has been found in the genome of *Astroviridae, Picornaviridae, Caliciviridae*, and *Coronaviridae* (30, 31). The exact role of the s2m element remains obscure. DAstV-5 possesses two NLS sequences. In contrast, the other avastroviruses possess only one NLS sequence. The NLS sequence mediates the process of viral protein entry into the nucleus, which influences virus replication and controls the host immune system (32). Therefore, we hypothesized that the two NLSs in DAstV-5 enhance the entry of virus proteins into the nucleus compared with the single NLS in other avastroviruses. However, the function of the two NLSs in DAstV-5 requires further study.

Although DAstV-3 CPH shares the lowest ORF2 amino acid genetic distance with the JM strain, the results demonstrated that these two strains are closely related to each other, but they belong to different genotypes. The JM strain shares genetic distances of 0.596–0.695 with the three official *Avastrovirus* species, indicating that JM could be classified as a novel genotype species in the *Avastrovirus* genus. Furthermore, JM shares genetic distances of 0.402–0.662 with all the other known unassigned avastroviruses, revealing that it represents an additional unassigned avastrovirus. Therefore, we designated strain JM as DAstV-5. The phenomenon that GAstVs cause gout in ducks and chickens demonstrates the cross-species transmission ability of goose astrovirus. However, the cross-species transmission ability of DAstV-5 is uncertain. The pathogenicity of DAstV-5 to chickens and geese should be further evaluated. This study recorded the first identification of novel duck astrovirus. Further investigation should be made to evaluate the pathogenicity and the infection status of DAstV-5 in ducks in China.

## Conclusion

In conclusion, we identified a novel duck astrovirus in ducklings suffering from visceral gout, namely DAstV-5 JM strain. We used virome sequencing and analysis to obtain the complete genome sequence of the virus. Based on the phylogenetic analysis and pairwise comparisons, the DAstV-5 JM strain was identified as a novel genotype species of avastrovirus. Our research enhances the understanding of the epidemiology and ecology of astroviruses in ducks.

## Data Availability Statement

The datasets presented in this study can be found in online repositories. The names of the repository/repositories and accession number(s) can be found below: https://www.ncbi.nlm.nih.gov/genbank/ OM095382.

## Ethics Statement

Ethical review and approval were not required for the study because the samples used in the present study were taken from field animals by veterinarians. The samples were taken and submitted to Institute of Animal Health, Guangdong Academy of Agricultural Sciences.

## Author Contributions

ML and MS supervised this project and designed the experiments. JZ, YH, LL, JD, and RK collected samples and performed experiments. JZ analyzed data and prepared manuscript. All authors read and approved the final manuscript.

## Funding

This work was supported by the Modern Agricultural Industrial Technology System Innovation Team of Guangdong Province (2020KJ137), the Special fund for scientific innovation strategy construction of high level Academy of Agriculture Science (R2020PY-JC001, 202110TD, R2020PY-JX014, R2020QD-049), the National Natural Science Foundation of China (No. 32102691), Guangdong Basic and Applied Basic Research Foundation (2021A1515012071), Guangzhou Basic and Applied Basic Research Foundation (202102021162), the Innovation Fund of Guangdong Academy of Agricultural Sciences-Industrial Special Project (202144), Guangzhou Science and Technology Plan project (201906040005), and Science and Technology Plan Projects of Guangdong Province (Grant No. 2021B1212050021), and the Collaborative Innovative Center of Guangdong Academy of Agricultural Sciences (XT202207).

## Conflict of Interest

The authors declare that the research was conducted in the absence of any commercial or financial relationships that could be construed as a potential conflict of interest.

## Publisher's Note

All claims expressed in this article are solely those of the authors and do not necessarily represent those of their affiliated organizations, or those of the publisher, the editors and the reviewers. Any product that may be evaluated in this article, or claim that may be made by its manufacturer, is not guaranteed or endorsed by the publisher.
