# Supplementary material for: First Identification and Genetic Characterization of a Novel Duck Astrovirus in Ducklings in China
Source: Front Vet Sci. 2022 Apr 8;9:873062. doi: 10.3389/fvets.2022.873062 (PMC9024104; doi:10.3389/fvets.2022.873062)
Supplement: Supplementary file 1 [file Table_1.DOCX]

Supplementary Material

**Supplementary Table 1｜**Five astrovirus segments identified by virome sequencing and analysis of DAstV-5 JM strain

| Segments identified by sequencing | Length | Most similar strain | Most similar sequence ID | Alignment similarity (%) | DNA or RNA |
| --- | --- | --- | --- | --- | --- |
| JMduck\|contig_420 | 1006 bp | Duck astrovirus CPH strain | KJ020899.1 | 73.60 | RNA |
| JMduck\|contig_708 | 685 bp | Chicken astrovirus GA2011 strain | JF414802.1 | 70.20 | RNA |
| JMduck\|contig_817 | 661 bp | Chicken astrovirus CAstV/CHN/HBLP717-1/2018 strain | MN725025.1 | 61.60 | RNA |
| JMduck\|contig_1460 | 253 bp | Duck astrovirus CPH strain | KJ020899.1 | 77.00 | RNA |
| JMduck\|contig_1756 | 395 bp | Turkey astrovirus strain TAstV/MI/01 | EU143846.1 | 52.00 | RNA |

**Supplementary Table 2｜**The primers we used to detect other viruses

| Virus | Primer sequences (5′-3′) | Product size (bp) |
| --- | --- | --- |
| GAstV | ATCGCAAAATGCAGGAGA | 342 |
|  | GTCCATTAGCTCCATAAAG |  |
| ARV | TAGCCACACCCGTGCTAGGA | 850 |
|  | ATAGAGGTRTTRATACCACG |  |
| DTMUV | GCAACCAGGCAAAGAGGT | 567 |
|  | AAGTGAGCCTCATCCATAAT |  |
| NDPV | GAGCATCAACTCCCGTATGTCC | 661 |
|  | CTACTTCCTGCTCGTCCGTGA |  |
| DCV | CGACTTATGTTATCTTTGGGCGT | 218 |
|  | GTCGTCCATGACGACTACGTCA |  |
| GPV | CAAGCATCAACTGTGGCACC | 589 |
|  | TCTGATCTACCCGGACAGCA |  |
| DEV | ATGTCTAATTCTGGAGGTTC | 354 |
|  | TTATCGCTGATCGTCTG |  |
| DAdV | GACATGGGGGCAACCTATTT | 745 |
|  | ATGATGTCCCGTCATCACT |  |
| AIV | GGGAGCAAAAGCAGGAGT | 1413 |
|  | GGAGTAGAAACAAGGAGTTTTTT |  |
| NDV | GACAAAGCAGTCAACATATACACC | 493 |
|  | TGAGTTCTACACCAACCTGCT |  |

**Supplementary Table 3｜** Primers used for the amplification of the gaps and for the verifying the complete genome sequence of JM

| Sequence name | Forward primer (5'-3') | Reverse primer (5'-3') | Forword primer position in JM | Reverse primer position in JM | Product size (bp) |
| --- | --- | --- | --- | --- | --- |
| duck astro-Gap 1 | AGTCTTCAACTTTCAGGGTGT | GTTGCCCACTCACTGCCATC | 66-86 | 1843-1862 | 1797 |
| duck astro-Gap 2 | GAACAGTTTGAAATGGAGACTAATG | TATTGTGCCATCAAATCTAGTCCAG | 2269-2293 | 4259-4283 | 2015 |
| duck astro-Gap 3 | AATGTAGGGAGGACCGAAAGTAG | GTTTGTCCTAACTCCTATTGG | 5014-5036 | 5590-5610 | 597 |
| duck astro-Gap 4 | ATAATGAATGTGCCCGATGG | GTTTGGTTCCCACTGTCC | 5914-5933 | 7228-7245 | 1332 |
| duck astro-Seq 1 | GCTTTTTCTAGCTTAGATGCACGC | ACTCTTCTCCAAAAGCCTCAAC | 1-24 | 1030-1051 | 1051 |
| duck astro-Seq 2 | TGTGTCGCCCGTTTTACATTCTT | GTTGCCCACTCACTGCCATC | 901-923 | 1843-1862 | 962 |
| duck astro-Seq 3 | TCTGGAATGAGGGAGTTAGTG | TATTCCTCTTCTGACAACATTTT | 1430-1450 | 2542-2564 | 1135 |
| duck astro-Seq 4 | GAACAGTTTGAAATGGAGACTAATG | TCATCTTGATCCCATCCTCG | 2269-2293 | 3672-3691 | 1423 |
| duck astro-Seq 5 | GCAGCGTGTAAACGCAAGAATG | TATTGTGCCATCAAATCTAGTCCAG | 3307-3328 | 4259-4283 | 977 |
| duck astro-Seq 6 | AAAGAAGATAGCGGATAATGACA | GTGCTACTTTCGGTCCTCCCTAC | 4043-4065 | 5017-5039 | 997 |
| duck astro-Seq 7 | AGAAATCCAACAAGGAAACTACC | TAAAGCAGAACCATCGGGC | 4818-4840 | 5925-5943 | 1126 |
| duck astro-Seq 8 | AGTGTTCACAGGCTCGCT | AAAAGCTCAAAAACTTAAGG | 5763-5780 | 7453-7472 | 1710 |

Primers used for the amplification of the gaps: duck astro-Gap 1–4. Primers used for the verifying the complete genome sequence of JM: duck astro-Seq 1–8.
